# Bedside Ultrasound Evaluation Uncovering a Rare Urological Emergency Secondary to Neurofibromatosis

**DOI:** 10.5811/westjem.2015.6.27585

**Published:** 2015-10-20

**Authors:** Thomas M. Nappe, Leonel Diaz, Elizabeth M. Evans

**Affiliations:** University of South Florida Morsani College of Medicine, Tampa, Florida. Lehigh Valley Hospital, Department of Emergency Medicine, Allentown, Pennsylvania

## CASE

A 56-year-old female presented to the emergency department (ED) with a chief complaint of urinary retention and overflow incontinence for 24 hours, preceded by progressive difficulty with voiding, worsening lower abdominal discomfort and bloating. Her past medical history was significant for small bowel obstruction and neurofibromatosis with an associated benign pelvic tumor that caused similar symptoms as a child, but had been known to be stable since that time. She had also recently been treated for a urinary tract infection. Her physical exam revealed tachycardia and a diffusely tender abdomen with a palpable, tender suprapubic mass extending just above her umbilicus.

Bedside ultrasonography was performed to visualize her kidneys and bladder and revealed bilateral hydronephrosis and a distended bladder with marked wall thickening (video), yielding further evaluation with computed tomography (CT). A urinary catheter was placed and 1,850 milliliters of urine were collected. CT of the abdomen and pelvis then confirmed bilateral hydronephrosis and a severely enlarged bladder with a diffusely thickened wall, consistent with the nodular appearance expected with neurofibroma of the bladder, as demonstrated in the figure. Laboratory analysis of urine and blood supported suspicion of urinary tract infection and obstructive uropathy, respectively. Histopathological analysis subsequently confirmed the presence of a neurofibroma.

## DISCUSSION

Neurofibroma of the bladder is an extremely rare manifestation of neurofibromatosis type 1, or von Recklinghausen disease, not typically seen in the ED.^1^,^2^ Although the bladder is the most commonly affected site within the genitourinary system, there are less than 80 reported cases in the literature.^2^ As in our patient, neurofibroma of the bladder can lead to obstructive uropathy with hydronephrosis.^3^ Here, we used bedside ultrasonography in the ED for evaluation of symptomatology, which led to the preliminary diagnosis of this rare manifestation, further captured by CT.

## Figures and Tables

**Figure 1:**
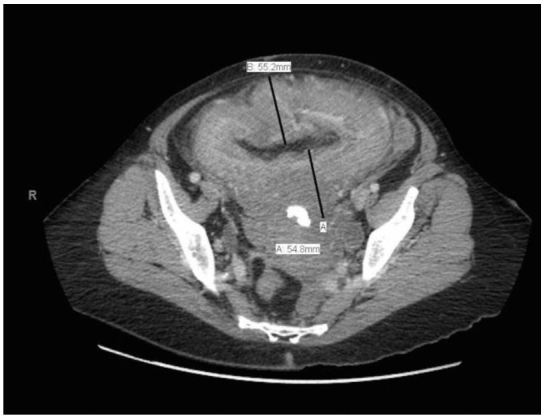
Computed tomography of enlarged, nondistended urinary bladder with diffusely thickened wall shown in transverse view after placement of a urinary catheter.

**Figure 2:**
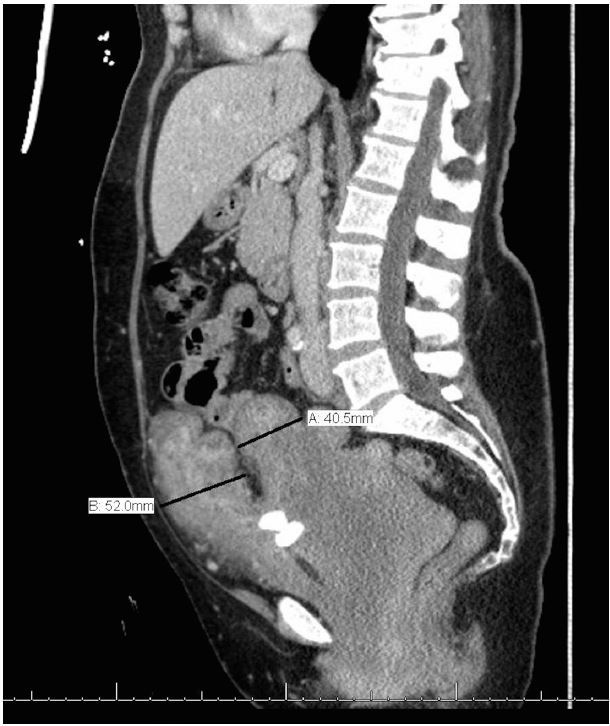
Computed tomography of enlarged, nondistended urinary bladder with diffusely thickened wall shown in sagittal view after placement of a urinary catheter.

**Video:** Ultrasound of enlarged, distended urinary bladder with diffusely thickened wall and resultant hydronephrosis, prior to placement of urinary catheter. Average bladder wall thickness is 2mm with distention.^4^
